# EHMTI-0211. Post-dural puncture headache related to spinal anesthesia for elective knee surgery

**DOI:** 10.1186/1129-2377-15-S1-C10

**Published:** 2014-09-18

**Authors:** JM Chung, JK Kim, KI Park, JY Kim, HA Yu

**Affiliations:** 1 Neurology, Inje University Seoul Paik Hospital, Seoul, Korea; 2 Orthopedic surgery, Inje University Seoul Paik Hospital, Seoul, Korea

## Introduction

Post-dural puncture headache (PDPH) has many risk factors for its development. However, it is not clear whether those factors are still significant in controlled procedural setting.

## Aims

To ascertain 1) risk factors for developing PDPH, and 2) clinical profiles of PDPH in the patients received knee surgery under spinal anesthesia(SA).

## Methods

We evaluated the presence of PDPH in all consecutive 400 patients (243 males, 157 females, age: 1-79 years) who received knee surgery under SA during 1 year at our hospital. SA was performed by one anesthesiologist with 25-G Quincke needle. Data regarding demographic profile, presumptive risk factors for PDPH, and clinical features of PDPH were analysed.

## Results

The incidence of PDPH was 6.8%(27 out of 400), which was higher in female(10.8%, p=0.01). Lower BMI, previous recurrent headache, and smoking were also identified as risk factors for PDPH. Duration of anesthesia or operation, age, or perioperative blood pressure did not differ for the patients with or without PDPH. Most of PDPH(77.7%) developed at least 6 hours past the end of operation. PDPH was usually orthostatic (74.1%) and started within 15 minutes on upright position. Nausea(66.7%) was the most frequent associated symptom. Headache intensity was variable. All PDPH were relieved spontaneously without epidural blood patch.

## Conclusions

PDPH related to elective knee surgery under SA was more frequent in patients of female, lower BMI, recurrent headache history, and smoker. PDPH patients showed benign clinical outcomes, which might be due to controlled procedural settings different from diagnostic lumbar puncture.

No conflict of interest.

